# Increased Maternal and Cord Blood Betatrophin in Gestational Diabetes

**DOI:** 10.1371/journal.pone.0131171

**Published:** 2015-06-26

**Authors:** Natalia Wawrusiewicz-Kurylonek, Beata Telejko, Mariusz Kuzmicki, Angelika Sobota, Danuta Lipinska, Justyna Pliszka, Beata Raczkowska, Pawel Kuc, Remigiusz Urban, Jacek Szamatowicz, Adam Kretowski, Piotr Laudanski, Maria Gorska

**Affiliations:** 1 Department of Endocrinology, Diabetology and Internal Medicine, Medical University of Bialystok, Bialystok, Poland; 2 Department of Gynecology, Medical University of Bialystok, Bialystok, Poland; 3 Department of Perinatology, Medical University of Bialystok, Bialystok, Poland; Medical University Innsbruck, AUSTRIA

## Abstract

**Aim:**

The aim of the study was to compare maternal and cord blood levels of betatrophin – a new peptide potentially controlling beta cell growth - as well as in its mRNA expression in subcutaneous adipose tissue, visceral adipose tissue and placental tissue obtained from pregnant women with normal glucose tolerance (NGT) and gestational diabetes (GDM).

**Methods:**

Serum betatrophin and irisin concentrations were measured by ELISA in 93 patients with GDM and 97 women with NGT between 24 and 28 week of gestation. Additionally, maternal and cord blood betatrophin and irisin, as well as their genes (*C19orf80* and *Fndc5)* expression were evaluated in 20 patients with GDM and 20 women with NGT at term.

**Results:**

In both groups, serum betatrophin concentrations were significantly higher in the patients with GDM than in the controls (1.91 [1.40-2.60] ng/ml vs 1.63 [1.21-2.22] ng/ml, p=0.03 and 3.45 [2.77-6.53] ng/ml vs 2.78 [2.16-3.65] ng/ml, p=0.03, respectively). Cord blood betatrophin levels were also higher in the GDM than in the NGT group (20.43 [12.97-28.80] ng/ml vs 15.06 [10.11-21.36] ng/ml, p=0.03). In both groups betatrophin concentrations in arterial cord blood were significantly higher than in maternal serum (p=0.0001). Serum irisin levels were significantly lower in the patients with GDM (1679 [1308-2171] ng/ml) than in the healthy women between 24 and 28 week of pregnancy (1880 [1519-2312] ng/ml, p=0.03). Both *C19orf80* and *Fndc5* mRNA expression in fat and placental tissue did not differ significantly between the groups studied.

**Conclusions:**

Our results suggest that an increase in maternal and cord blood betatrophin might be a compensatory mechanism for enhanced insulin demand in GDM.

## Introduction

Betatrophin, also known as lipasin [1, 2], atypical angiopoietin like protein 8 (ANGPTL8) [3], refeeding induced fat and liver protein (RIFL) [4] and chromosome 19 open reading frame 80 (C19orf80), is a secreted protein of 198 amino acids, primarily expressed in the liver and adipose tissue [3, 4, 5]. Betatrophin was shown to promote pancreatic beta-cell proliferation, expand beta-cell mass and improve glucose tolerance in an animal model of insulin resistance caused by an insulin receptor antagonist S961 infusion [5]. However, recently Gusarova et al. [6] reported that Angptl8(-/-) mice underwent entirely normal beta cell expansion in response to insulin resistance resulting from either a high-fat diet or from the administration of S961 peptide, and that overexpression of ANGPTL8 did not change beta cell growth nor glucose metabolism in experimental animals. Moreover, Jiao et al. [7] found that elevated mouse hepatic betatrophin expression did not increase human β-cell replication in the transplant setting. Betatrophin was also described as a liver enriched nutritional regulator that inhibits lipoprotein lipase and reduces triglyceride (TG) clearance [2]. Actually, knockdown of the betatrophin gene leads to impaired adipogenesis [4] and reduced TG content [8], whereas betatrophin overexpression increases circulating TG concentration [2, 6]. Experimental studies have shown that betatrophin expression is suppressed by starving [2, 3] and induced by food intake [2, 3, 9], insulin [4] and an exposure to a cold environment [1]. Moreover, recently Zhang et al [10] reported that betatrophin gene (*Gm6484/C19orf80*) expression in primary rat adipocytes and 3T3-L1-derived adipocytes may be up-regulated by irisin—a novel myokine and adipokine [11, 12] encoded by *Fndc5* (fibronectin type III domain containing 5) gene, which induces browning of white adipose tissue, thereby increasing total body energy expenditure and improving glucose tolerance in experimental animals [11]. However, the impact of betatrophin on insulin secretion and glucose homeostasis seems still far from clear. Furthermore, clinical studies have shown increased [9, 13–18], but also unchanged [19] or even decreased [20] circulating betatrophin levels in patients with obesity and type 1 or type 2 diabetes in comparison with healthy individuals.

Since gestational diabetes mellitus (GDM) is regarded to be a prediabetic state, characterized by an enormous insulin resistance and inadequate insulin compensation [21], we hypothesized that betatrophin levels may be increased in women with GDM as an adaptive mechanism enhancing beta-cell proliferation and insulin secretion. Moreover, we aimed to find out whether there are any differences in betatrophin (*C19orf80*) and irisin (*Fndc5*) mRNA expression in subcutaneous adipose tissue (SAT), visceral adipose tissue (VAT) and placental tissue obtained from pregnant women with normal glucose tolerance (NGT) and GDM.

## Materials and Methods

### Study population

The present study was a continuation of our previous investigations concerning the role of pro-and anti-inflammatory cytokines and adipokines in GDM, supported by the State Committee for Scientific Research (grant No. KBN 2 P05E 08829 and N N407 141937) [22–25]. The population studied consisted of 93 patients with GDM and 97 women with NGT, matched for age, gestational age and BMI, recruited from the gynaecological out-patient clinic of the Medical University of Bialystok (Group 1), as well as 20 patients with GDM and 20 women with NGT, who delivered healthy, singleton infants at term, undergoing elective Caesarean section at the Department of Perinatology, Medical University of Bialystok (Group 2). Women with multiple pregnancy, pre-existing glucose intolerance, pregnancy-induced hypertension, preeclampsia, acute or chronic inflammation and active smokers were not included. All patients underwent a 75 g oral glucose tolerance test (OGTT) in the 24th– 30th week of gestation and GDM was diagnosed according to the WHO (2013) criteria [26]. All patients from Group 2 were treated with diet only. The patients from Group 1 were invited for a control visit 10–12 weeks after childbirth and 45 patients with prior GDM, as well as 39 women with NGT during pregnancy were available 3 months postpartum. Written informed consent was obtained from all participants before enrolment, and the protocol was approved by the local ethics committee (Medical University of Bialystok, an approval number R-I-002/20/2009).

### Analytical methods

In Group 1, blood samples were collected after an overnight fast, before glucose load. In Group 2, maternal blood samples were collected in the fasting state, before anaesthesia was give, whereas umbilical cord blood samples were taken immediately after delivery. Plasma glucose concentration was measured by enzymatic method with hexokinase (Cobas c111, Roche Diagnostics Ltd, Switzerland). Serum insulin and C-peptide levels were assayed by immunoradiometric method (DiaSource Europe SA, Belgium) and glycated haemoglobin (HbA1c) was evaluated by a high performance liquid chromatography technique (BIO-RAD Laboratories, Germany). The following indices of insulin sensitivity and insulin secretion were calculated: HOMA-IR = FPG [mmol/l] x FPI [μU/ml]/22.5, HOMA-β [%] = 20 x FPI [mU/l]/FPG [mmol/l]-3.5, the Matsuda and de Fronzo index (IS_OGTT_) = 10,000/√ [(FPG x FPI) x (G x I)], where FPG = fasting plasma glucose, FPI = fasting plasma insulin, G = mean glucose and I = mean insulin during the OGTT [27], and the disposition index (DI_120_) = IS_OGTT_ x AUC_Ins120_/AUC_Glu120_, where AUC_Ins120_/AUC_Glu120_ = the ratio of the area under the insulin curve (AUC) to glucose AUC during 0–120 min of the OGTT) [28]. Total cholesterol, HDL-cholesterol and TG concentrations were measured by enzymatic methods (ANALCO-GBG, Poland). LDL-cholesterol concentration was calculated using the Friedewald equation. Serum betatrophin concentration was determined using a commercially available human ELISA kit (USCN Life Science Inc., China) with both an intra- and an inter-assay coefficient of variation (CV) <10%. Serum irisin level was measured using commercial ELISA (BioVendor, Czech Republic) with an intra-assay CV of 6.5% and an inter-assay CV of 8.8%.

### Tissue samples

Subcutaneous adipose tissue adjacent to the lower abdominal incision and visceral (omental) adipose tissue samples were obtained from 20 patients with GDM and 20 women with NGT during elective Caesarean section. Placental tissue was sampled after manual removal of the placenta from approximately 5mm deep incision from the fetal side of the placenta. All samples were immediately immersed into RNAlater (Qiagen) and stored at -80°C until assayed.

### RNA extraction and cDNA synthesis

Total RNA was isolated and purified using RNeasy Lipid Tissue Mini Kit (Qiagen, Germany) following the manufacturer’s protocol. Total RNA concentration was determined using NanoDrop ND-1000 spectrophotometer (NanoDrop 1000, THERMO Scientific, USA). cDNA synthesis was performed using High Capacity cDNA Reverse Transcription Kit (Life Technologies, USA) in the MJ Research Thermal Cycler (Model PTC-200, USA).

### Quantitative real-time RT-PCR

The expression of betatrophin *(C19orf80)*, irisin *(Fndc5)* and uncoupling protein 1 (*UCP-1*) genes was measured by quantitative real-time PCR, using commercially designed TaqMan Gene Expression Assays (Hs00218820_m1, Hs00222453_m1 and Hs00401006_m1, respectively, Applied Biosystem, USA) and the 7900HT Fast Real Time PCR System (Life Technologies, USA). Glyceraldehyde-3-phosphate dehydrogenase (GAPDH) gene was used as an endogenous control. The reaction mixture consisted of 5μl cDNA, 10 μl of TaqMan Gene Expression Master Mix (Applied Biosystem, USA) and 2μl of each TaqMan Gene Expression Assay. The thermal profile of the reaction was as follows: 2 min at 50°C, 10 min at 95°C, followed by 40 cycles of 15 sec at 95°C and 1 min at 60°C. Data were analyzed by RQ Manager Software (Life Technologies, USA).

### Statistical analysis

The differences in gene expression between the groups studied were calculated by REST (Relative Expression Software Tool) 2009 (www.gene-quantification.info) [29]. Other data were analyzed by the STATISTICA 10.0 for Windows Software (StatSoft.Inc, Tulsa, USA). Before analysis, data were tested for normality of distribution using the Shapiro-Wilk test. Differences between the groups were compared by Mann-Whitney U test and relationships between variables were tested by Spearman’s correlations. Wilcoxon signed rank test was used to compare betatrophin and irisin concentrations in maternal and cord blood, as well as their levels during and after pregnancy. Finally, multiple linear regression analysis was applied to establish the factors independently associated with serum betatrophin and irisin, as well as their genes expression in fat and placental tissue. P value less than 0.05 was regarded as statistically significant. Data were shown as medians with interquartile ranges.

## Results

### Characteristics of the groups studied

In Group 1. the patients with GDM had significantly higher fasting (p<0.0001) and post-load glucose levels (p<0.0001), fasting (p = 0.02) and 120 min. post-load insulin (p<0.0001), HOMA-IR (p = 0.0009), HbA1c (p = 0.001) and triglyceride concentration (p = 0.004), as well as lower HOMA-β (p = 0.001), DI_120_ (p<0.00001) and IS_OGTT_ (p<0.00001) than had the patients with NGT (Table 1). Three months postpartum, the patients with previous GDM had still higher post-load glucose concentrations in comparison with the healthy women (p = 0.02, Table 2). The patients with GDM from Group 2 had significantly higher HbA1c values than had the women with NGT (p = 0.001, Table 3).

**Table 1:** Clinical characteristics of Group 1 during pregnancy.

	NGT	GDM	P value
**n**	97	93	
**Age (years)**	30 (28–34)	31.5 (28–35)	0.22
**Parity**	1 (1–2)	2 (1–3)	0.12
**Gestational age (week)**	28 (26–30)	28 (26–30)	0.55
**Pregestational BMI (kg/m** ^**2**^ **)**	23.6 (21.9–26.9)	24.7 (21.7–29.3)	0.18
**Current BMI (kg/m** ^**2**^ **)**	27.1 (24.8–30.1)	28.4 (24.8–32.2)	0.20
**Weight gain (kg)**	8.0 (6.5–11.0)	9.0 (5.6–11.0)	0.95
**Fasting glucose (mmol/l)**	4.4 (4.2–46)	4.8 (4.4–5.2)	<0.00001
**Glucose 30’ (mmol/l)**	7.2 (6.7–7.9)	9.1 (8.3–9.5)	<0.00001
**Glucose 60’ (mmol/l)**	7.3 (6.2–8.2)	10.3 (9.7–11.0)	<0.00001
**Glucose 120’ (mmol/l)**	6.1 (5.5–6.5)	8.6 (7.8–9.2)	<0.00001
**Fasting insulin (pmol/l)**	93.1 (68.8–125.7)	108.3 (80.6–150.0)	0.02
**Insulin 30’ (pmol/l)**	539.1 (402.1–742.2)	477.5 (361.8–722.0)	0.27
**Insulin 60’ (pmol/l)**	498.4 (388.4–1055.1)	762.3 (478.0–1018.1)	0.19
**Insulin 120’ (pmol/l)**	456.5 (352.9–603.3)	756.5 (523.7–1056.2)	<0.00001
**HOMA-IR**	2.71 (1.87–3.71)	3.37 (2.41–4.50)	0.0009
**HOMA-β (%)**	295.2 (206.7–410.7)	245.8 (168.2–338.6)	0.001
**IS**_**OGTT**_	3.95 (3.28–5.16)	2.76 (1.89–4.0)	<0.00001
**DI**_**120**_	319.3 (245.2–389.7)	210.1 (166.0–270.3)	<0.00001
**HbA1c (%)**	4.8 (4.4–5.2)	5.1 (4.8–5.3)	0.001
**Total cholesterol (mmol/l)**	6.1 (5.5–6.9)	6.4 (5.6–7.1)	0.22
**HDL-cholesterol (mmol/l)**	1.9 (1.6–2.2)	1.8 (1.6–2.1)	0.40
**LDL-cholesterol (mmol/l)**	3.2 (2.6–4.2)	3.4 (2.5–4.3)	0.89
**Triglycerides (mmol/l)**	2.0 (1.6–2.5)	2.3 (1.8–3.0)	0.004

Data are shown as medians (interquartile range), NGT, normal glucose tolerance, GDM, gestational diabetes mellitus, differences between NGT and GDM groups were analyzed by Mann-Whitney U test.

**Table 2:** Clinical characteristics of Group 1 three months postpartum.

	NGT	GDM	P value
**n**	39	45	
**Age (years)**	33 (29.5–34.5)	31 (29–36)	0.89
**Current BMI (kg/m** ^**2**^ **)**	25.3 (23.0–27.9)	24.3 (21.9–28.0)	0.97
**Fasting glucose (mmol/l)**	4.8 (4.5–5.1)	5.0 (4.5–5.3)	0.36
**Glucose 30’ (mmol/l)**	7.2 (6.3–8.3)	8.1 (7.2–9.1)	0.02
**Glucose 60’ (mmol/l)**	6.5 (5.5–8.0)	7.7 (6.6–9.0)	0.02
**Glucose 120’ (mmol/l)**	4.9 (4.4–5.7)	5.7 (4.7–6.8)	0.02
**Fasting insulin (pmol/l)**	86.2 (47.8–16.4)	73.0 (47.3–108.3)	0.96
**Insulin 30’ (pmol/l)**	300.0 (238.1–582.9)	353.8 (228.0–533.5)	0.61
**Insulin 60’ (pmol/l)**	288.4 (184.1–428.0)	396.2 (209.4–734.3)	0.18
**Insulin 120’ (pmol/l)**	210.4 (145.0–406.1)	226.3 (164.9–407.7)	0.76
**HOMA-IR**	2.69 (1.35–3.4)	2.56 (1.41–3.4)	0.98
**HOMA-β (%)**	162.6 (128.0–212.9)	182.1 (105.3–246.0)	0.97
**IS**_**OGTT**_	5.64 (3.92–8.70)	5.12 (3.87–7.12)	0.46
**DI**_**120**_	238.6 (170.5–347.5)	231.4 (170.1–273.3)	0.37
**HbA1c (%)**	5.3 (5.0–5.5)	5.3 (5.0–5.6)	0.74
**Total cholesterol (mmol/l)**	4.4 (3.9–5.3)	4.7 (4.3–5.4)	0.35
**HDL-cholesterol (mmol/l)**	1.7 (1.5–1.9)	1.6 (1.3–1.8)	0.51
**LDL-cholesterol (mmol/l)**	2.3 (1.8–3.0)	2.7 (2.2–3.2)	0.12
**Triglycerides (mmol/l)**	1.0 (0.7–1.5)	0.8 (0.61.1)	0.08
**Birth weight (g)**	3300 (2950–3600)	3525 (3000–3700)	0.41

Data are shown as medians (interquartile range), NGT, normal glucose tolerance, GDM, gestational diabetes mellitus, differences between NGT and GDM groups were analyzed by Mann-Whitney U test.

**Table 3:** Clinical characteristics of Group 2.

	NGT	GDM	P value
**n**	20	20	
**Age (years)**	31 (29.5–33)	32 (27.5–36)	0.44
**Parity**	2 (1–2)	2 (1–2)	0.82
**Gestational age (week)**	39 (38–40)	39.5 (39–40)	0.42
**Pregestational BMI (kg/m** ^**2**^ **)**	22.7 (20.9–27.7)	25.6 (19.6–31.2)	0.92
**Current BMI (kg/m** ^**2**^ **)**	29.7 (26.8–34.5)	31.6 (24.8–35.9)	0.52
**Weight gain (kg)**	16.5 (8.5–19.5)	13.5 (11.5–19.5)	0.71
**Fasting glucose (mmol/l)**	4.1 (3.9–4.4)	4.1 (3.7–4.4)	0.69
**Fasting insulin (pmol/l)**	65.9 (50.0–81.9)	59.0 (51.4–89.6)	0.77
**Fasting C-peptide (pmol/ml)**	0.60 (0.34–0.74)	0.53 (0.36–0.80)	0.93
**HOMA-IR**	1.65 (1.35–2.10)	1.52 (1.20–2.30)	0.88
**HbA1c (%)**	4.8 (4.5–5.1)	5.1 (4.8–5.3)	0.001
**Total cholesterol (mmol/l)**	7.2 (6.1–8.5)	6.9 (6.2–8.2)	0.76
**HDL-cholesterol (mmol/l)**	1.6 (1.3–2.0)	1.7 (1.4–2.1)	0.41
**LDL-cholesterol (mmol/l)**	3.6 (3.2–4.6)	3.5 (2.7–3.9)	0.36
**Triglycerides (mmol/l)**	3.7 (3.3–4.6)	3.7 (2.9–4.3)	0.73
**Birth weight (g)**	3285 (3120–4125)	3530 (3350–4000)	0.28
**Cord blood glucose (mmol/l)**	3.2 (2.9–3.6)	3.2 (2.8–3.5)	0.76
**Cord blood insulin (pmol/l)**	50.0 (41.0–59.0)	49.3 (34.7–66.6)	0.68
**Cord blood C-peptide (pmol/ml)**	0.18 (0.15–0.26)	0.33 (0.15–0.40)	0.05
**Cord blood HOMA-IR**	0.98 (0.88–1.35)	1.02 (0.84–1.24)	0.85

Data are shown as medians (interquartile range), NGT, normal glucose tolerance, GDM, gestational diabetes mellitus, differences between NGT and GDM groups were analyzed by Mann-Whitney U test.

### Maternal and cord blood betatrophin and irisin

#### Group 1

**Fig 1:**
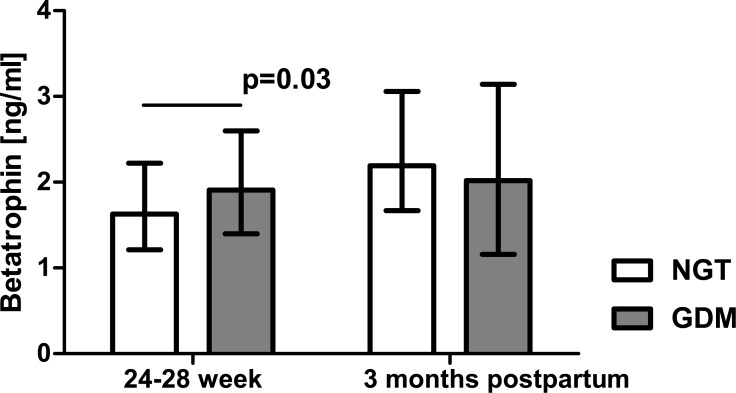
Serum betatrophin concentration in patients with normal glucose tolerance (NGT) and gestational diabetes (GDM) from Group 1; data were analyzed by Mann-Whitney U test and shown as medians with interquartile ranges.

**Fig 2:**
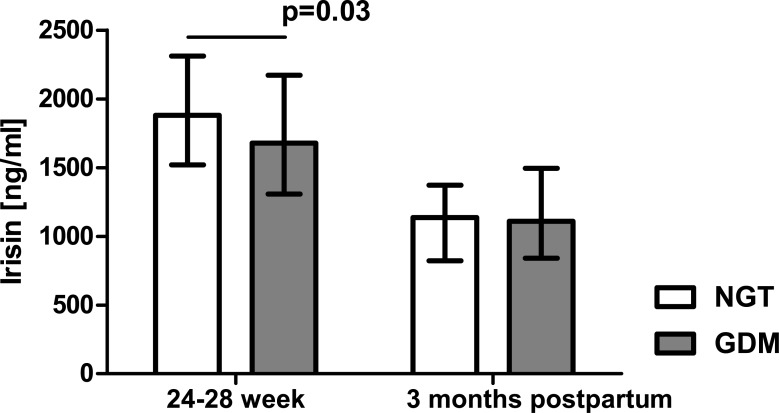
Serum irisin concentration in patients with normal glucose tolerance (NGT) and gestational diabetes (GDM) from Group 1; data were analyzed by Mann-Whitney U test and shown as medians with interquartile ranges.

Serum betatrophin concentration in the patients with GDM was significantly higher than in the healthy women (1.91 [1.40–2.60] ng/ml vs 1.63 [1.21–2.22] ng/ml, p = 0.03, Fig 1). In contrast, serum irisin levels were markedly lower in the patients with GDM as compared with the NGT group (1679 [1308–2171] ng/ml vs 1880 [1519–2312] ng/ml, p = 0.03, Fig 2). Three months postpartum, neither betatrophin nor irisin levels differed significantly between the women with prior GDM and NGT (2.02 [1.16–3.14] ng/ml vs 2.19 [1.67–3.06] ng/ml and 1109 [841–1495] ng/ml vs 1137 [822–1372] ng/ml, respectively, Figs 1 and 2).

In the whole group studied serum betatrophin concentration correlated positively with triglyceride level (R = 0.15, p = 0.03), whereas irisin was negatively associated with fasting glucose concentration (R = -0.231, p = 0.003) and positively with HOMA-β (R = 0.189, p = 0.03). In the subgroup with NGT, serum irisin also correlated negatively with fasting glucose level (R = -0.223, p = 0.04). Three months postpartum in the subgroup with prior GDM, betatrophin concentration correlated positively with 120 min. post-load glucose level (R = 0.373, p = 0.02). Multiple regression analysis revealed that serum betatrophin concentration during pregnancy was independently predicted by fasting glucose (β = -0.251, p = 0.02) and the diagnosis of GDM (β = 0.194, p = 0.04, R^2^ = 0.08), whereas none of the variables studied (the patient’s age, gestational age, diagnosis of GDM, BMI, glucose, insulin, total cholesterol, HDL-cholesterol and triglyceride concentrations) predicted betatrophin concentration 3 months postpartum. Serum irisin was significantly associated only with fasting glucose, both during pregnancy (β = -0.314, p = 0.007, R^2^ = 0.03) and 3 months after childbirth (β = -0.387, p = 0.03, R^2^ = 0.04).

#### Group 2

**Fig 3:**
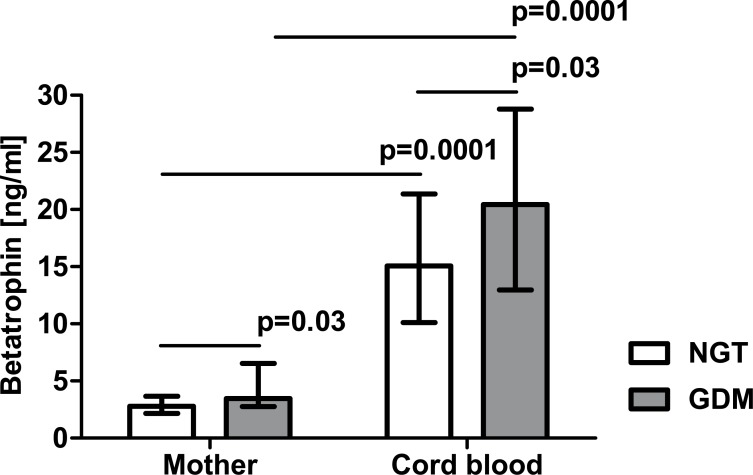
Serum betatrophin concentration in patients with normal glucose tolerance (NGT) and gestational diabetes (GDM) from Group 2; data were analyzed by Mann-Whitney U test and shown as medians with interquartile ranges.

**Fig 4:**
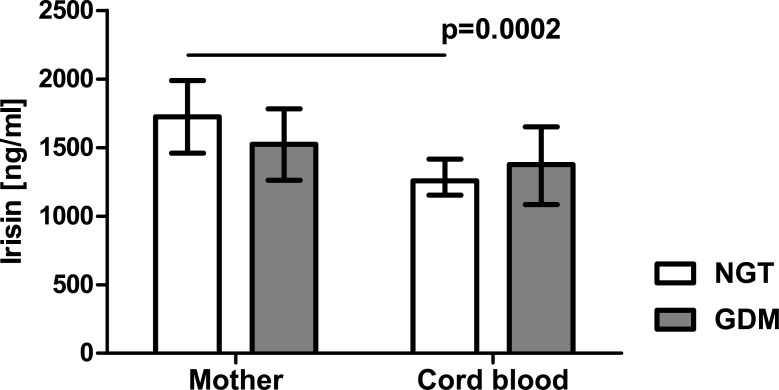
Serum irisin concentration in patients with normal glucose tolerance (NGT) and gestational diabetes (GDM) from Group 2; data were analyzed by Mann-Whitney U test and shown as medians with interquartile ranges.

Both maternal and cord blood betatrophin levels were significantly higher in the patients with GDM than in the group with NGT (3.45 [2.77–6.53] ng/ml vs 2.78 [2.16–3.65] ng/ml, p = 0.03 and 20.43 [12.97–28.80] ng/ml vs 15.06 [10.11–21.36] ng/ml, p = 0.03, respectively, Fig 3), whereas maternal and cord blood irisin levels did not differ significantly between the two groups (1524 [1261–1783] ng/ml vs 1723 [1460–1988] ng/ml and 1375 [1084–1652] ng/ml vs 1257 [1153–1415] ng/ml, respectively, Fig 4). Betatrophin concentrations in arterial cord blood were significantly higher than in maternal serum (p = 0.0001 in both groups), whereas irisin levels in cord blood were lower in comparison with maternal serum concentrations (p = 0.0002 in the NGT group).

In the whole group studied and in the subgroup with GDM, maternal betatrophin concentration correlated negatively with serum C-peptide level (R = -0.43, p = 0.005 and R = -0.46, p = 0.04, respectively). In the patients with GDM, there was a positive correlation between maternal and cord blood betatrophin level (R = 0.50, p = 0.03). In the women with NGT, maternal betatrophin correlated positively with serum TG concentration (R = 0.49, p = 0.04), whereas cord blood betatrophin level was significantly associated with maternal insulin (R = 0.48, p = 0.03) and HOMA-IR (R = 0.52, p = 0.02). Cord blood irisin correlated significantly with maternal irisin level, both in the whole group studied (R = 0.50, p = 0.0015) and in the subgroups with GDM (R = 0.55, p = 0.02) and NGT (R = 0.50, p = 0.02). In all the patients studied and in the subgroup with NGT, there were also negative correlations between cord blood irisin and cord blood insulin (R = -0.34, p = 0.03 and R = -0.54, p = 0.01, respectively). Multiple regression analysis revealed that maternal betatrophin was associated with serum C-peptide level (β = -0.462, p = 0.03) and the diagnosis of GDM (β = 0.447, p = 0.04, R^2^ = 0.13). Cord blood betatrophin level was significantly associated with the presence of GDM (β = 0.39, p = 0.04), explaining 10% of its variability. Serum irisin concentration was not related to any of the variables studied, whereas cord blood irisin was predicted by maternal irisin level (β = 0.52, p = 0.04, R^2^ = 0.04).

### *C19orf80*, *Fndc5* and *UCP-1* mRNA expression in fat and placental tissue

*Betatrophin* (*C19orf80* ***)***, *Fndc5* and *UCP-1* mRNA were detected in all samples of SAT, VAT and placental tissue. *C19orf80* and *Fndc5* mRNA expression in fat and placental tissue did not differ significantly between the two groups studied, whereas *UCP-1* mRNA expression in the placenta was higher in the patients with GDM than in the women with NGT (p = 0.02, Table 4). The highest expression of *C19orf80* and *Fndc5* mRNA was detected in SAT and the lowest in the placental tissue (p<0.00001), whereas *UCP-1* mRNA expression was significantly higher in VAT than in SAT and the placenta (p<0.00001).

**Table 4:** Fold changes of relative gene expression in subcutaneous adipose tissue, visceral adipose tissue and placental tissue from women with gestational diabetes in comparison with healthy pregnant women.

Gene	SAT		VAT		Placenta	
	ExpR	p-value	ExpR	p-value	ExpR	p-value
***C19orf80***	1.33	0.50	1.12	0.79	1.02	0.95
***Fndc5***	1.34	0.40	1.02	0.94	1.36	0.40
***UCP-1***	0.51	0.16	0.92	0.87	**1.88**	**0.02**

Relative gene expression of the respective gene in the healthy pregnant group = 1.

ExpR, expression ratio; SAT, subcutaneous adipose tissue, VAT, visceral adipose tissue, differences between groups were calculated by REST 2009 Software.

In the whole group studied and in the subgroup with GDM, there were negative correlations between *Fndc5* mRNA expression in VAT and gestational age (R = -0.47, p = 0.005 and R = -0.61, p = 0.01, respectively). No association between serum betatrophin or irisin and their mRNA expression in fat and placental tissue was found in both groups studied. Multiple regression analysis revealed that *C19orf80* mRNA expression in SAT was related to gestational age (β = 0.46, p = 0.04, R^2^ = 0.31), whereas its expression in VAT and placental tissue was not associated with any of the variables analyzed. *Fndc5* mRNA expression in all tissues studied and *UCP-1* mRNA expression in SAT were not related to any of the variables included to the model. *UCP-1* mRNA expression in VAT was significantly predicted by the patient’s age (β = -0.46, p = 0.04), gestational age (β = 0.67, p = 0.009), maternal glucose (β = 0.48, p = 0.04) and insulin level (β = -0.5, p = 0.02), together explaining 30% of its variability, whereas *UCP-1* mRNA expression in placental tissue was significantly associated with the presence of GDM (β = -0.38, p = 0.04) and BMI (β = -0.48, p = 0.02), together explaining 26% of its variability.

## Discussion

Our study showed that serum betatrophin concentration was significantly higher in the patients with GDM than in the pregnant women with NGT. Moreover, multiple regression analysis revealed that the diagnosis of GDM was an independent predictor of serum betatrophin level. Notably, in the group of women at term, maternal betatrophin concentration correlated negatively with serum C-peptide, which might suggest that decreased capacity for insulin secretion may enhance circulating betatrophin. This finding seems to be in line with the results of Tokumoto et al. [30], who reported that in patients with type 2 diabetes, plasma betatrophin levels inversely correlated with insulin secretion capacity, measured as an increment of C-peptide concentration in response to glucagon stimulation. Our results are also consistent with the finding of elevated betatrophin in patients with obesity and type 2 diabetes [9, 13–18], however unchanged [19] or even decreased [20] serum betatrophin levels have been reported in diabetic or/and insulin resistant patients as well. Interestingly, although we could not directly compare betatrophin concentrations between the patients in the 24th– 30th week of pregnancy and these at term, it seems that circulating betatrophin raises during pregnancy and is much higher in the 3rd than in the 2nd trimester. Yi et al. [5] observed that in pregnant mice serum betatrophin concentrations were 20 times higher than in non-pregnant animals, but this has never been studied in humans. Nevertheless, our results might suggest that augmented insulin resistance and enormous demands for insulin in patients with GDM may lead to an increased production of betatrophin as a booster of beta-cell proliferation and insulin secretion. Although an elevated level of betatrophin in the GDM group was not related to an increase in its mRNA expression in fat or placental tissue, there is good evidence that the liver is the main source of circulating betatrophin in humans [3, 4, 5]. Furthermore, our study showed that not only maternal but also cord blood betatrophin levels were significantly higher in the patients with GDM in comparison with the NGT group, possibly as a compensatory mechanism for enhanced insulin demands in the face of hyperglycaemic environment. Noteworthy, betatrophin concentration in cord blood was about five times higher than in maternal serum, which might suggest its role in promoting beta-cell proliferation during intrauterine life. However, it should be mentioned that recently Gusarova et al [6] questioned the ability of betatrophin to control beta cell expansion and suggested that ANGPTL8 plays a key role in lipid metabolism, and in particular in directing fatty acids to adipose tissue for storage in the fed state [6, 8]. In our study circulating betatrophin correlated positively with serum triglyceride level, which is in line with the results of experimental studies, demonstrating a reduced TG clearance and an increase in circulating TG as an effect of betatrophin overexpression [2].

In the present study circulating irisin levels were significantly lower in the patients with GDM than in the healthy women between 24 and 30 week of gestation, which is consistent with our previous report [31]. However, in the subgroup of women at term serum and cord blood irisin concentrations did not differ significantly between the patients with and without GDM. Conversely, Ebert at al. [32] did not find significant differences in circulating irisin between the women with GDM and NGT in the 24th– 28th week of pregnancy, whereas Yuksel et al. [33] demonstrated markedly lower irisin concentrations in the patients with GDM as compared with healthy pregnant women at term. Our results seem to be in line with the findings of Piya et al. [34], who reported no significant differences in serum irisin levels between the non-obese, obese and GDM subjects at term. However, after adjusting for BMI, lipids and glucose, the lowest values were noted in the non-obese healthy women [34]. Aforementioned discrepancies might be explained by different characteristics of the groups studied, different time of sampling or/and various methods used for the assay, however further investigations on irisin in pregnancy seem to be needed. Moreover, both in the present study and in the only other report concerning cord blood irisin concentration in GDM [33], there were no significant differences between the cases and the controls. Notably, although maternal irisin increases during pregnancy, probably as an adaptive mechanism for enhanced insulin or/and irisin resistance [31, 32], the placenta does not seem to be a substantial source of circulating irisin [32, 35]. Furthermore, it was demonstrated that *Fndc5* expression in SAT was approximately 100 times lower than in muscle [12]. The present study also showed that *Fndc5* expression in fat and placental tissue was relatively low and did not differ significantly between the women with and without GDM. On contrary, Kurdiova et al [12] demonstrated that *Fndc5* expression in adipose tissue obtained from patients with impaired glucose tolerance and type 2 diabetes was much lower than in healthy individuals. Interestingly, the present study showed that *UCP-1* mRNA expression in the placenta was significantly higher in the patients with GDM than in the healthy pregnant women, but the role of this “browning” gene, primarily expressed in adipose tissue and up-regulated by irisin [10, 11], in human placenta seems unknown.

In conclusion, our study showed that both maternal and cord blood betatrophin levels were significantly higher in the patients with GDM as compared with healthy pregnant women, possibly as a compensatory mechanism for increased insulin resistance and enormous insulin demand.
